# Motor features of abstract verbs determine their representations in the motor system

**DOI:** 10.3389/fpsyg.2022.957426

**Published:** 2022-08-30

**Authors:** Xiang Li, Dan Luo, Chao Wang, Yaoyuan Xia, Hua Jin

**Affiliations:** ^1^Academy of Psychology and Behavior, Tianjin Normal University, Tianjin, China; ^2^Faculty of Psychology, Tianjin Normal University, Tianjin, China; ^3^Department of Psychology, Xinxiang Medical University, Xinxiang, China; ^4^Faculty of Education, Henan Normal University, Xinxiang, China; ^5^Department of Physical Education, Zhejiang University of Finance and Economics, Hangzhou, China

**Keywords:** embodiment, motor features, representation, action–sentence compatibility effect, abstract verbs, motor system

## Abstract

Embodied cognition theory posits that concept representations inherently rely on sensorimotor experiences that accompany their acquisitions. This is well established through concrete concepts. However, it is debatable whether representations of abstract concepts are based on sensorimotor representations. This study investigated the causal role of associated motor experiences that accompany concept acquisition in the involvement of the motor system in the abstract verb processing. Through two experiments, we examined the action–sentence compatibility effect, in the test phase after an increase in motor features during the learning phase for abstract verbs with low motor features (Experiment 1) or novel words with no conceptual features at all (Experiment 2). After associated motor experiences were added in the word learning phase, action–sentence compatibility effect was found in the semantic processing tasks during the test phase for abstract verbs (Experiment 1a) and novel words (Experiment 2). This was lacking in the word font color judgment task requiring no semantic processing (Experiment 1b). Coupled with our previous study, these findings suggest that motor features formed during word learning could causally affect embodiment in the motor system for abstract verbs, and reactivation of motor experiences in abstract verb processing depends on a given task’s demands. Our study supports the view that conceptual representations, even abstract concepts, can be grounded in sensorimotor experiences.

## Introduction

Embodied cognition theory states that human concepts are represented in the sensorimotor and emotional systems that are engaged by corresponding experiences during concept acquisition, and comprehending words comprises reactivating the same experiences that are responsible for perceiving and acting upon these concepts (Barsalou, 1999, 2008; Gallese and Lakoff, 2005; Pulvermüller et al., 2005; Jirak et al., 2010; Kiefer and Pulvermuüller, 2012; Borghi et al., 2017; Buccino et al., 2019). While the involvement of motor and somatosensory systems in concrete concept processing is well established in both behavioral and neuroimaging literature (Hauk et al., 2004; Marino et al., 2013; Su et al., 2013; García et al., 2019; Klepp et al., 2019; Zappa et al., 2019; Dreyer et al., 2020; Zhao et al., 2020; Togato et al., 2021), it is still debated whether abstract concepts are represented in the sensorimotor system. The main challenge for embodiment is to provide convincing explanations and evidence of the embodiment of abstract concepts (Barsalou, 2008; Buccino et al., 2019; Ostarek and Huettig, 2019).

Taking verbs as an example, abstract concepts such as *think* lack a direct connection to sensorimotor experiences in contrast to concrete concepts such as *kick*. It is unclear whether representations of abstract verbs involve the sensorimotor system. Few studies have explored the involvement of the sensorimotor system in the processing of abstract verbs. This is perhaps due to the prolonged bias toward the meanings of concrete words in this domain (Muraki et al., 2020a) and preference for nouns of the widely used semantic ratings, such as concreteness ratings (Brysbaert et al., 2014). Although several behavioral (Glenberg and Kaschak, 2002; Schaller et al., 2017; van Dam and Desai, 2017) and neuroimaging studies (Guan et al., 2013; Sakreida et al., 2013; Moreno et al., 2015; Ghio et al., 2016; Tian et al., 2020; meta-analysis see: Yang and Shu, 2016) have discussed motor simulation in terms of comprehending sentences or phrases, including abstract verbs, their conclusions cannot be directly applied to single abstract verb processing. The linguistic structures, syntactic or pragmatic effects, or contextual information might affect simulations in abstract verb processing (Barsalou et al., 2008; Yang and Shu, 2016; Conca et al., 2021a). For instance, a functional magnetic resonance imaging (fMRI) study found that involvement of the sensorimotor areas in idiomatic action sentences (e.g., *The congress is* ***grasp****ing at straws in the crisis*) is less than in the literal ones (e.g., *The instructor is* ***grasp****ing the steering wheel very tightly*) including the same verb (Desai et al., 2013; see also: Raposo et al., 2009; Romera Lauro et al., 2013). Xue et al. (2015) showed the sensorimotor sentential context effect on subsequent body–object interaction word processing. Zwaan (2021) proposed that “one cannot extrapolate one linguistic unit to another.” Therefore, it is necessary to examine sensorimotor representations of abstract verbs at the lexical level.

Among studies that provided the early neuroimaging evidence supporting embodied views, which mainly focused on concrete action verbs, some focused on abstract verbs and yielded inconsistent results (van Dam et al., 2010; Rodríguez-Ferreiro et al., 2011; Moseley et al., 2012; Papeo et al., 2012; Dalla Volta et al., 2014; Innocenti et al., 2014; Tomasino et al., 2014, 2018). For instance, Tomasino et al. investigated the neural substrates related to the mental simulation of abstract (e.g., *to think*) and action verbs (e.g., *to grasp*) during a mental imagery task and a letter detection task in adults (Tomasino et al., 2014) and adolescents (Tomasino et al., 2018). Both studies did not find any activation in sensorimotor-related areas during the processing of abstract verbs, but there was activation in the right supramarginal gyrus and other regions. Thus, the authors suggested that abstract verbs may be mostly represented in emotional and social networks rather than the sensorimotor system. Nevertheless, other findings have provided evidence that abstract verbs may be represented in the sensorimotor system. Papeo et al. (2012) found that abstract verb reading (e.g., *to adore*) after a mental rotation task based on motor strategy, compared with reading after a rotation task based on visuospatial strategy, could increase activities in the left primary motor cortex, the bilateral premotor cortex, and the right somatosensory cortex. These brain regions were the same as those activated while reading hand action-related verbs (e.g., *to stir*).

The semantic content of abstract concepts is rich and heterogeneous, and the differential relevance of the sensorimotor, introspective, linguistic, affective, and social experiences has been validated for different types of abstract concepts (Harpaintner et al., 2018; Villani et al., 2019; Lynott et al., 2020). Dreyer and Pulvermüller (2018) proposed that the variance of previous results regarding involvement of the sensorimotor system in the processing of abstract meaning might be explained by the different inclusion criteria for abstract stimuli. Previous studies overlooked differences in modality-specific features (e.g., *emotional feature or motor feature*) when selecting abstract verbs. Additionally, some recent studies have found that abstract words with modality-specific features show differences in reaction time or neural activities (for behavioral studies, see: Vonk et al., 2019; Jin and Li, 2022; Muraki et al., 2020a; for neuroimaging studies, see: Moseley et al., 2012; Harpaintner et al., 2020; Muraki et al., 2020b; Conca et al., 2021b). For example, Muraki et al. (2020a,b) investigated behavioral and neural differences in a go/no-go syntactic classification task among abstract verbs, that is, emotional abstract verbs (e.g., *annoy*), psychological abstract verbs (e.g., *accept*), and non-embodied abstract verbs (e.g., *allow*). Their findings suggest that abstract concepts rely on complex representations in sensorimotor, affective, social, and linguistic systems.

Therefore, it can be speculated that the involvement of the motor system in the representation of abstract verbs is modulated by motor features. Abstract verbs with more direct motor experiences acquired during their learning are more likely to be predominant in motor features and grounded much more in the motor system. Thus, it is easier to observe embodied effects when these verbs are processed. Recent studies have provided indirect evidence for this hypothesis. Öttl et al. (2017) and Günther et al. (2018) proved that direct sensorimotor experiences associated with novel word learning are necessary for reactivating experiential traces, at least during noun processing. Moreover, using fMRI, Harpaintner et al. (2020) found that the relative predominance of motor features in abstract nouns significantly predicts the amplitude of estimated neural activation in sensorimotor-related areas. However, whether motor features play a causal role in sensorimotor representations of abstract verbs remains unknown.

Jin and Li’s study has preliminarily proved that involvement of the motor system in abstract verb processing is related to the motor features of abstract verbs *per se* (Jin and Li, 2022). Their first experiment involved a syntactic classification task for concrete verbs (e.g., *throw*), abstract verbs (e.g., *abandon*), and filler words (e.g., *tombstone*), and they found the action–sentence compatibility effect during concrete verb processing, but not during abstract verb processing. The ACE has been validated as evidence for the interaction between action language processing and motor behaviors, which is assumed to indicate semantic processing in the brain’s motor system (Glenberg and Kaschak, 2002; Klepp et al., 2019). This means that when the implicit direction of the language stimuli and the movement direction of required responses are congruent (i.e., both toward or away from the participant), participants’ responses to the verbs are significantly faster than when the two directions are incongruent. In their next experiment, the authors further examined the ACE in processing abstract verbs with high or low motor features (e.g., *bear* vs. *reveal*) in the same task. It was observed only during the processing of abstract verbs with high motor features, and its magnitude was significantly correlated with the motor features of abstract verbs. These findings, in line with the embodied views for representations of abstract concepts, provided evidence for the abovementioned speculation, i.e., the involvement of the motor system in abstract verb processing largely depends on the degree of predominance of the motor features of abstract verbs. However, it is noteworthy that in Jin and Li’s (2022) study, all stimuli were old words that the participants were familiar with. This makes it difficult to provide causal evidence for the relationship between the predominance of motor features of abstract verbs and their representations in the motor system.

As Ostarek and Huettig (2019) stated, “evidence for a causal link between simulation and language comprehension must be the gold standard for assessing embodied accounts.” Therefore, in this study, we artificially added motor experiences to abstract verbs with low motor features (Experiment 1) and novel words without endogenous or inherent conceptual features (Experiment 2) during the learning phase through self-performed hand actions to highlight their motor features. The primary goal was to investigate the causal role of motor features in the involvement of the motor system in abstract verb processing and to provide direct evidence for the embodied explanations of abstract verbs. Previous studies found that meaning-congruent motor actions could improve word learning (Casasanto and de Bruin, 2019; Repetto et al., 2021), and imagined body movements facilitated the comprehension of metaphorical phrases (Wilson and Gibbs, 2007), suggesting that appropriate motor actions may better enhance the encoding of words into long-term memory. Therefore, to successfully increase the predominance of motor features in the abstract verbs, in the learning phase of both experiments, the participants were instructed to study the words and perform a specific hand movement toward or away from themselves. The ACE was adopted as the embodied effect to indicate motor simulation during abstract verb processing. Specifically, Experiment 1 needed participants to memorize abstract verbs with low motor features while performing direction-congruent hand actions. It examined the ACE in the test phase in the syntactic classification task (Experiment 1a) and the word font color judgment task (Experiment 1b). Experiment 2, with almost identical learning procedures as Experiment 1a, used created meaningless words (novel words) and instructed participants to judge whether a novel word was semantically consistent or inconsistent with the real word preceding it.

Following grounded theories, we hypothesized that after the learning phase, wherein the motor features of either abstract verbs (Experiment 1) or novel words (Experiment 2) were increased, the processing of the learning words would elicit greater motor simulation to exhibit the ACE in the testing task. However, semantic processing is not automatic, and embodied language processing may depend on tasks (Ostarek and Huettig, 2019). Thus, we expected that the ACE would only appear in the test phase of Experiments 1a and 2, but not in that of Experiment 1b. Our study, from a causal perspective, would demonstrate that it is the predominance of motor features in abstract verbs that determines how much abstract verbs are grounded in the motor system.

## Experiment 1

### General materials and methods: experiments 1a and 1b

#### Participants

In both experiments, the participants were recruited from a university in China and received a financial reimbursement of 60 RMB/h. All were right-handed native Chinese speakers. Written informed consent was obtained from all participants. To ensure adequate power, both experiments maintained a sample size above the recommended value (i.e., 24) obtained through a statistical power analysis (G*Power 3.1.9.2, Faul et al., 2007) with a medium effect size (0.25) and medium power (1–β = 0.8).

In Experiment 1a, 32 undergraduates volunteered to participate (four males); the mean age was 19.94 years (SD = 1.46). Another group of 34 undergraduates participated in Experiment 1b, but one participant’s data were removed because she did not complete the entire experiment. The mean age of the remaining 33 participants was 20.00 years (SD = 1.41) (four males).

#### Materials

In both Experiments, 24 Chinese abstract verbs (e.g., 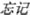/*forget*) were used as target stimuli: 12 for learning and 12 for non-learning, and 24 nouns were used as filler stimuli (the full set of verbal stimuli is provided in Supplementary Material). The 24 filler nouns were presented only in Experiment 1a. All the words had two characters. These verbs were selected from abstract verbs with low motor features in Jin and Li’s (2022) Experiment 2. It is important to note that the ACE was not found in the original study during the processing of these abstract verbs. These verbs were evaluated by undergraduates on a seven-point Likert scale to measure the weight of motor features or concreteness (Jin and Li, 2022) (1 = very low weight of motor features or very low level of abstraction; 7 = very high weight of motor features or very high level of concreteness). The average scores for the learning verbs on motor features and concreteness were 2.23 (SD = 0.31) and 3.09 (SD = 0.72), respectively. Additionally, half of these verbs implied an action direction toward the body, while the other half implied an action direction away from the body. The average ratings in the implicit direction for the “toward” and “away” verbs were significantly different (toward vs. away: 2.17 ± 0.26 vs. 5.62 ± 0.63, *p* < 0.001; 1 refers to direction toward the body and 7 refers to direction away from the body) (Jin and Li, 2022). Each learning verb was printed in black and bold Song font, with a font size of 36 on one side of white-background cards (9*9 cm^2^).

For non-learning verbs, the average ratings of the six “toward” and six “away” verbs were significantly different (toward vs away: 2.43 ± 0.31 vs. 5.38 ± 0.61, *p* < 0.001). The average ratings of the motor features and concreteness were 2.24 (SD = 0.35) and 3.79 (SD = 0.64), respectively.

#### Procedure

Each participant individually performed a learning phase preceded by a test phase in a quiet laboratory setting. The experimenter was present throughout the experiment to ensure that instructions were followed. The procedures of Experiments 1a and 1b were identical in the learning phase, but differed in the test phase.

##### Learning phase

**FIGURE 1:**
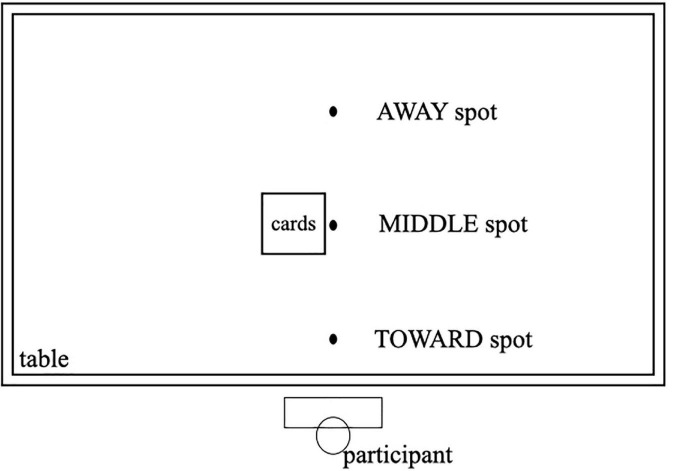
Illustration of the layout for the three spots on the experimental table.

Three equidistant spots, namely, TOWARD, MIDDLE, and AWAY spots, were marked vertically in a line on the table. The distance between the spots was 25 cm. The AWAY spot was above the center and far away from the participant, whereas the TOWARD spot was below the center and closer to the participant (see Figure 1).

Initially, all the cards were placed on the MIDDLE spot. Participants were instructed to memorize the verbs on the cards while thinking about whether the implicit direction of the verb meaning was away from or toward themselves. Thereafter, they had to place the card with their right hand on either the AWAY spot or TOWARD spot in accordance with the word’s implicit direction. It should be noted that the direction-thinking instruction was designed to ensure the same deep semantic encoding of motor features in the verbs among all participants. Each card was shown to memorize for 4 s (timed by a timer). Every fourth seconds, the timer beeped as a cue to remind participants to place the current card in the right place and begin to memorize the next one. They memorized the cards, one at a time, in a random order. When through with all 12 cards, the experimenter confirmed that all cards were placed correctly, shuffled the cards, and placed them back on the MIDDLE spot for the next round of learning. Each card was shown for learning five times. Four additional cards were prepared to familiarize the participants with the procedure. The entire learning phase took approximately 15 min.

To ensure that all participants were in agreement with the implicit action directions of the 12 learning verbs, each participant was asked to have a quick look at a piece of paper displaying the verbs and their implicit directions before learning. In Experiments 1a and 1b, none of the participants had different opinions about the directions.

##### Test phase

After the learning phase, the participants performed a task using a computer. Different tasks were used in Experiments 1a and 1b. In both tasks, the keyboard, used as the response device, was rotated 90° counterclockwise and placed on the right side of the computer screen such that the three keys, namely, A, G, and L, were naturally aligned vertically to the participant. The distance between A and G was the same as that between L and G, with G in the middle, while A was near the participant and L was far.

**FIGURE 2:**
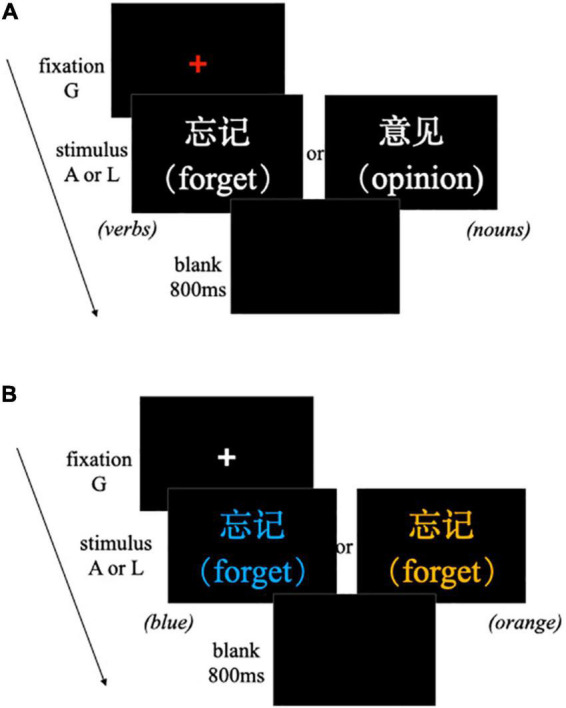
Procedure of an experimental trial in the test phase. **(A)** Experiment 1a; **(B)** Experiment 1b.

Experiment 1a used the syntactic classification task wherein the participants were instructed to decide whether a word on the screen was a verb. If the word was a verb, they were instructed to press the L key; if it was not a verb, they pressed A. The pressing keys were counterbalanced across the participants. The stimuli were presented in the Song font, with a font size of 40, in white letters on a black background using E-Prime 2.0 (Psychology Software Tools, Pittsburgh, PA, United States). Each trial began with a fixation cross. Once the participant pressed G, a word replaced the cross and disappeared until the participant made a response. Subsequently, after an 800-ms blank screen, the next trial began (see Figure 2A). The participants were instructed to respond as quickly and accurately as possible and press keys with the same finger (e.g., the index finger) of their right hand during the entire task. The word stimuli in the task included 12 learning abstract verbs and 12 non-learning abstract verbs with low motor features and 24 filler nouns, resulting in a total of 48 trials. Prior to the formal test, the participants exercised eight additional practice trials to familiarize themselves with the task. The entire testing phase of Experiment 1a took approximately 15 min.

Experiment 1b was a word font color judgment task (see Figure 2B). In this task, the word stimuli were presented in one of four colors (red, blue, orange, or purple), and the participants had to respond to the color of the words. If the font color of the word was red or blue, they pressed L; if it was orange or purple, they pressed A. Two colors were mapped to each response direction to increase the difficulty of the task and engage participants in slightly longer processing of the stimuli before making their response (Öttl et al., 2017; Günther et al., 2018). The assignment of colors to A or L responses was balanced between the participants. The word stimuli included 12 learning abstract verbs and 12 non-learning abstract verbs. Each word was presented once in each of the four colors. The entire task consisted of 96 trials (24 words × 4 colors). The participants exercised eight additional practice trials to familiarize themselves with the task. The other procedures and requirements were identical to those of Experiment 1a. The participants completed the test phase of Experiment 1b within approximately 10 min.

#### Design

Both experiments employed a 2 learning (abstract verbs for learning vs. non-learning) × 2 congruency (the implicit direction of the verb and the movement direction of the required response in the test task were congruent vs. incongruent) within-subject design. The dependent variable was participants’ reaction time (RT).

### Results and discussion

Only the RT for the correct target trials was analyzed. The RT that exceeded the mean by more than three standard deviations was treated as an outlier to ensure the lowest proportion of elimination data (Marmolejo-Ramos et al., 2015) and to be consistent with recent studies on the embodiment of language (e.g., Muraki et al., 2020a; Zhao et al., 2020). The significance criterion for all analyses was set at α = 0.05. All analyses were performed using IBM SPSS 20.

#### Experiment 1a

**FIGURE 3:**
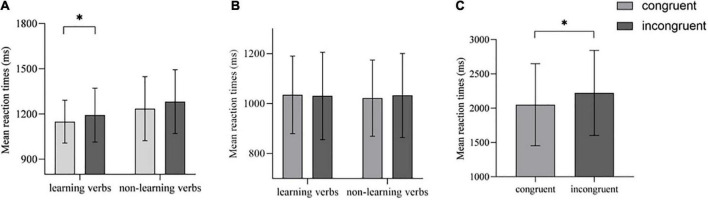
Mean reaction times for each condition in the experiments. **(A)** Experiment 1a; **(B)** Experiment 1b; **(C)** Experiment 2. Error bar represents ± 1SD. * indicates *p* < 0.05.

Incorrect and outlier trials resulted in a loss of 22 trials (2.86%) in the syntactic classification task of Experiment 1a. The RT for learning and non-learning verbs was averaged for each participant in each condition (congruent vs. incongruent). The Shapiro–Wilk test showed that the RT for the four conditions (*p*s > 0.05) had normal distributions. Thus, a repeated measures ANOVA was first performed on the data. The results showed that the main effects of learning [F(1, 31) = 30.611, *p* < 0.001, η^2^ = 0.497] and congruency [F(1,31) = 5.797, *p* = 0.022, η^2^ = 0.158] were significant. Specifically, the participants responded faster to the learning verbs than to the non-learning verbs, indicating a strong learning effect. And the participants responded faster in the congruent condition than in the incongruent condition. However, the interaction was not significant [F(1,31) < 1, *p* = 0.938]. To address our research questions, we further conducted simple effect analyses. For learning verbs, the RT of the congruent condition was significantly lower than that of the incongruent condition [1,149 ± 142 vs. 1,192 ± 179 ms, F(1,31) = 5.661, *p* = 0.024, η^2^ = 0.154], that is, the ACE was observed. For non-learning verbs, there was no significant difference in response time between the congruent and incongruent conditions [1,235 ± 212 vs. 1,282 ± 212 ms, F(1, 31) = 1.893, *p* = 0.179, η^2^ = 0.058] (see Figure 3A).

Thus, after increasing the motor features by associating motor experiences with verb learning in this experiment, a weak but typical ACE was found for abstract verbs that lacked the same in the previous study. Although the interaction was not significant, the main effect of learning was extremely significant.

Taken together, it is suggested that increasing the association between verbs and motor experiences could increase the motor features of abstract verbs, and the processing of abstract verbs would reactivate motor experiences. Accordingly, it is likely to observe the ACE of abstract verbs. At the same time, non-learning abstract verbs without an increase in motor features did not have an ACE.

However, it is important to note that the syntactic classification task used in Experiment 1a was a semantic task in which participants needed to process the given word’s meaning to complete the task. The next question was whether the reactivation of the motor experiences in abstract verb processing depended on the semantic demands of the task. Therefore, a word font color judgment task requiring no semantic processing was used in the test phase of Experiment 1b. This was done to further explore the possible influence of task demand on the causal link between the motor features of abstract verbs and their motor representations.

#### Experiment 1b

Incorrect and outlier trials resulted in a loss of 41 trials (2.6%) in the word font color judgment task of Experiment 1b. The RT for learning and non-learning verbs was averaged for each participant in each condition (congruent vs. incongruent). The Shapiro–Wilk test showed that the RT in the learning incongruent condition (0.874, *p* < 0.05) and non-learning congruent condition (0.919, *p* < 0.05) was not normally distributed, and a natural logarithmic transformation was applied to all data before the analysis. Thereafter, the logarithmic RT was subjected to repeated measures ANOVA. The results showed that the main effects of learning and congruency and the interaction between the two were not significant [F(1,32)s < 1, *p*s > 0.05] (see Figure 3B). The simple effect analyses also found no significant differences between the conditions [F(1,32)s < 2, *p*s > 0.05]. In other words, with identical manipulation in the learning phase, the ACE did not appear in the color judgment task, even for the learning verbs. The findings of Experiment 1b suggested that the reactivation of motor experiences in abstract verbs was not automatic, but was more likely to depend on the explicit semantic demands of the task.

Experiments 1a and 1b confirmed that increasing motor features in words could increase the involvement of the motor system in the semantic processing of abstract verbs. However, it is noteworthy that abstract verbs learned in Experiment 1 were familiar and meaningful to participants and may have been grounded in multiple experiences, such as linguistic, sensorimotor, and emotional experiences. Thus, it is difficult to determine whether the observed ACE was caused solely by an increase in motor features or by the interaction of increased motor features with other experiences. Moreover, using real words in Experiment 1 made it difficult to exclude the potential influence of other linguistic characteristics of the materials themselves on the results. One solution to this problem would be to add motor experiences to artificially created words without inherent conceptual features before learning and then to prove that these words exhibit the ACE.

Hence, to address the material deficiency of Experiment 1, Experiment 2 used artificially created words to further verify the causal role of motor features in the involvement of the motor system in abstract verb processing. If an ACE can be reliably found in the test phase, it would demonstrate that whether an abstract verb represented in the motor system depends on its motor features.

## Experiment 2

### Participants

The recruitment method and sample size of the participants were identical to those of Experiment 1. In Experiment 2, 30 undergraduates volunteered to participate (three males); the mean age was 20.23 years (SD = 1.14). All were right-handed native Chinese speakers. Written informed consent was obtained from all participants.

### Materials

Ten pronounceable novel words were created prior to the experiment. All novel words were made of two Chinese characters (e.g., 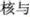/he yu), which did not carry any meaning as words. Before the formal experiment, 15 additional undergraduates evaluated the meaning, concreteness, familiarity, and motor features of the novel words. All novel words were meaningless, and the mean scores of the other three dimensions were all lower than 1.5 on a seven-point Likert scale. Ten abstract verbs with low motor features were selected randomly from the learning verbs of Experiment 1 as the corresponding interpretive words for the learning of novel words. Half of the words implied an action direction toward the body, while the other half implied an action direction away from the body (the mean ratings of the two directions were 2.21 ± 0.26 vs. 5.70 ± 0.68, respectively). The average ratings of the 10 abstract verbs on motor features and concreteness were 2.30 (SD = 0.28) and 3.22 (SD = 0.72), respectively. Five novel words were paired with “toward” verbs, whereas the remaining five were paired with “away” verbs. Pairings between novel words and abstract verbs were randomized and fixed among all participants during Experiment 2.

Similar to Experiment 1, a novel word and an abstract verb paired with it were printed on one side of a card and were connected by a dash in the middle (e.g., “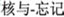/he yu- forget”). During the learning phase, these cards were initially located on the MIDDLE spot of the table, and then, the participants placed them toward or away from themselves, according to the instructions, as in Experiment 1.

### Procedure

The procedure was mostly the same as that in Experiment 1. The critical differences are described in the following sections.

#### Learning phase

In Experiment 2, the participants were instructed to learn and memorize the novel words and their interpretive word meanings through 10 card-printed word pairs for a subsequent test. While learning, they had to think about whether the implicit action direction of a novel word was toward or away from themselves and had to place the card on either the AWAY or TOWARD spot, one by one. Each card was learned four times, with 8 s provided for each time (timed by a timer). The other procedures and requirements were identical to those in Experiment 1. The entire learning phase took approximately 15 min.

#### Test phase

**FIGURE 4:**
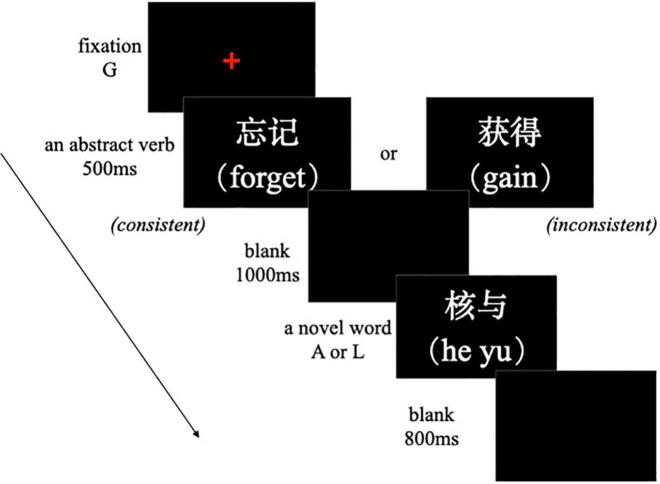
Procedure of an experimental trial in the test phase of Experiment 2.

A semantic task was programmed and run using E-Prime 2.0. The trial began with a fixation cross. When the G key was pressed, an abstract verb replaced the cross and was presented for 500 ms, which was followed by a 1,000-ms blank screen. Subsequently, a novel word appeared. Here, the participants needed to judge whether the novel word had the same meaning as the previously presented abstract verb and move their right hand to respond. If the answer was “yes,” they pressed A; if it was “no,” they pressed L, or vice versa. Once the response was provided, the novel word disappeared, and after an 800-ms blank screen, the next trial began (see Figure 4). Likewise, the response device was a keyboard rotated 90° counterclockwise on the right side of the computer screen. The participants were instructed to respond with the same finger (e.g., the index finger) of their right hand. The response keys were counterbalanced across participants.

All the word stimuli in the task were from the learning phase. Each novel word randomly appeared once with the original paired verb and once with the one of the remaining nine verbs. There were two experimental blocks, each containing 10 trials, with an equal number of semantically consistent and inconsistent trials. The other requirements were the same as those in Experiment 1. Prior to the official test, four practice trials were conducted to familiarize the participants with the procedure and response keys. The test phase of Experiment 2 took approximately 10 min.

### Design

A single-factor within-subject design with two levels (congruency: congruent vs. incongruent) was used in Experiment 2. The dependent variable was participants’ reaction time.

### Results and discussion

An accuracy rate in the test task of all the participants was 75.5% on average, which was above the chance level, indicating participants’ successful learning of novel words.

Only the correct RT for both semantically consistent and inconsistent trials was analyzed. After further eliminating the outliers (3SD, 11 trials), the mean RT of each participant was computed for each condition (congruent vs. incongruent). The Shapiro–Wilk test showed that the RT for both congruent and incongruent conditions had normal distributions (*p*s > 0.05). Subsequently, a paired sample *t*-test (one-tailed) was performed. The results revealed a significant difference between the two conditions. The RT of all participants in the congruent condition was significantly lower compared with the incongruent condition [2,050 ± 597 ms vs. 2,222 ± 620 ms, *t*(29) = −2.454, *p* = 0.010, *Cohen’s d* = 0.45] (see Figure 3C).

When the influence of possible confounding variables was excluded, the same congruency effect was obtained in Experiment 2. These findings demonstrated that even meaningless novel words could have an ACE after coupling motor experiences while learning. Experiment 2 further confirmed the causal role of motor features in the involvement of the motor system in the representation of abstract verbs.

## General discussion

This study artificially added motor experiences to abstract verbs with low motor features (Experiment 1) and novel words without inherent conceptual features (Experiment 2) during the learning phase. It did so to examine whether the involvement of the motor system in representations of abstract verbs depended on motor features by means of the ACE paradigm. In the two experiments, a weak but significant ACE was found in tasks that required semantic processing (Experiments 1a and 2), although the effect might be smaller than previously estimated. This may also be the reason why the interaction was not significant in Experiment 1a. These findings are consistent with our hypothesis mentioned in Introduction. However, the same embodied congruency effect did not appear in Experiment 1b, suggesting that the ACE, at least for abstract verbs, might be task-dependent.

### Motor features of abstract verbs causally affect their representations in the motor system

One major challenge for embodiment is to explain how abstract knowledge is represented in the brain (Buccino et al., 2019). According to Ostarek and Huettig (2019), the gold standard for assessing embodied views must be evidence for a causal link between embodied simulation and language comprehension. In the field of embodied language comprehension, this causal link has been fully discussed in empirical studies based on concrete concepts (Edmiston and Lupyan, 2017; Ostarek and Huettig, 2017; Öttl et al., 2017; Günther et al., 2018; Davis et al., 2020). For instance, Davis et al. (2020) conducted two experiments to provide partial support for the causal role of visual simulation in understanding words that were more experienced in the same (i.e., visual) modality. Both experiments showed that, when people made simple semantic judgments on words (i.e., to hear a word and judge “is this an animal or not?”), their responses were faster and more accurate, compared with responses when there was interference from a concurrent task condition, and the interference was scaled in proportion to the ratings of the corresponding modality. These results suggested that visual experiences determine the extent to which the concepts with high visual features are grounded in the visual system. Öttl et al. (2017) found a significant compatibility effect between the location of a word’s referent while learning and the moving direction of the response (upward/downward) to the color of the corresponding word in the test phase. Importantly, each artificial word learned in this study was randomly assigned to an object referent placed in the upper or lower location of a wall, thus providing direct evidence of the necessity of sensorimotor experiences for embodied language comprehension, at least during the learning of concrete contents.

As for empirical evidence related to the motor representations of abstract verbs, Glenberg et al. (2008) used single-pulse transcranial magnetic stimulation (TMS, a particularly useful neuroscientific method for probing causality in the motor domain, Ostarek and Huettig, 2019) to provide neurophysiological evidence for modulation of the motor system during the comprehension of sentences including abstract verbs. Experiment 1 replicated an ACE in the comprehension of Italian action sentences describing both concrete and abstract transfers. Using TMS, Experiment 2 found that when the TMS pulse was delivered 200 ms after the onset of the verb, the motor evoked potentials (MEPs) were larger than those delivered at the end of the sentences, and the MEPs to both concrete and abstract transfer sentences were greater than to the no-transfer sentences. This excluded the alternative explanation that the activity in the motor cortex during language comprehension found in Experiment 1 was simply a post-comprehension process.

However, as detailed in Introduction, as the results obtained at the sentence level cannot be extrapolated to those at the lexical level, the causal link between motor simulation and abstract verb processing still lacks of empirical evidence. In the field of conceptual metaphors, various studies have demonstrated that abstract concepts related to time (Santiago et al., 2007), social power (Schubert et al., 2009), or affective evaluation (Meier and Robinson, 2004) are mapped onto space (see Santiago et al., 2011, for a review). For instance, based on the association of “good” with “right” and “bad” with “left,” Casasanto and Chrysikou (2011) found that long-term or even a few minutes’ changes in motor fluency can reverse implicit associations between emotional valence and left/right space, showing the causal role of motor experience in abstract thought. While the focus of these studies was on the grounding of abstract concepts in sensorimotor experiences by means of metaphoric mappings, we were more interested in the specific factors that might modulate the involvement of the motor system in the representations of abstract verbs. Jin and Li’s (2022) found a correlation between involvement of the motor system in abstract verb processing and motor features of abstract verbs, and this study further confirmed the causal role of motor features in the involvement of the motor system in abstract verb processing. It also provided direct evidence for the embodied explanations of abstract concepts. Our findings showed that the predominance of motor features determined the extent of the representations in the motor system for abstract verbs. Similar to concrete concepts, representations of abstract verbs and sensorimotor experiences share neural substrates. The more motor experiences encoded during an abstract verb acquisition, the more dependent its representation is on the system of motion. Therefore, the inconsistent results in previous studies about the involvement of the motor system in abstract word processing might result from the difference in motor features of the abstract stimuli. Additionally, our results are consistent with reactivation theories of sensorimotor learning, which postulate that brain areas active during the encoding of words are reactivated when the same words are recalled (Masumoto et al., 2015; Repetto et al., 2021).

Furthermore, according to recent promising approaches within an embodied framework, abstract concepts rely on complex representations within not only sensorimotor and affective systems, but also linguistic, introspective, and social systems (Barsalou et al., 2008; Kousta et al., 2011; Borghi and Binkofski, 2014; Vigliocco et al., 2014; Borghi et al., 2019; Buccino et al., 2019). In studies that did not find involvement of the motor system in abstract verb processing (e.g., Dalla Volta et al., 2014; Innocenti et al., 2014; van Dam and Desai, 2017; Gijssels et al., 2018), the abstract stimuli may have had motor features too low to elicit effective activation of the motor system, as they may have been more represented in the affective, social, or linguistic systems (Tomasino et al., 2014, 2018; Vigliocco et al., 2014; Muraki et al., 2020a,b). Importantly, the ACE is an embodied effect that primarily indicates the functional role of the motor system in language comprehension and concept processing (Klepp et al., 2019; Morey et al., 2021). Using such an appropriate index to detect motor representations of abstract concepts, our results provide behavioral evidence for modality-specific representations (representations within modality-specific systems for perception, action, and emotion) of motor features of abstract verbs, similar to neuroimaging studies on concrete words (e.g., Hauk et al., 2004; Popp et al., 2019; Kuhnke et al., 2020). Thus, the degree of involvement of the motor system in the processing of abstract words may vary according to their dominant features of the abstract word. Recent studies have shown that conceptual processing involves both modality-specific sensorimotor areas and higher-level multimodal regions (Fernandino et al., 2016; Popp et al., 2019; Harpaintner et al., 2020; Kuhnke et al., 2020). Further research is required to provide neural evidence of the causal links between the dominant features in abstract concepts and their representations in modality-specific and multimodal regions.

### Reactivation of motor experiences in abstract verb processing depends on task demands

Based on the results of Experiment 1b, we further speculate that the reactivation of motor experiences in the abstract verb processing might not be automatic but dependent on task demands. If the task requires no semantic processing, similar to the word font color judgment in Experiment 1b, it is possible to not observe the ACE.

In fact, some existing studies demonstrated that sensorimotor recruitment during concrete knowledge processing is modulated by task demands (Papeo et al., 2009; Lebois et al., 2015; Shen et al., 2016; Ostarek and Huettig, 2017; Vukovic et al., 2017; Gálvez-García et al., 2020; Kuhnke et al., 2020). Ostarek and Huettig (2017) proved that low-level visual processing can be causally involved in language comprehension, but their recruitment is not automatic; it depends on the type of information required in a given task situation. Through three experiments, the authors found that visual noise interfered more with concrete words processing (relative to abstract words) but only in the concreteness task that required visual information to be accessed; this was the case not in the lexical decision task nor in the word class judgment task. Similar task-specific results were observed in Vukovic et al.’s (2017) study that assessed the role of motor areas in word processing. When repetitive transcranial magnetic stimulation (rTMS) of left motor cortex was delivered within 200 ms of word onset in the lexical decision task and the concreteness judgment task, only in the latter task the rTMS did slow down the behavioral response to action-related verbs and facilitate abstract verbs. Furthermore, in a recent electromyographic study, spontaneous arm muscle activation was observed only in tasks that required deep processing of manual verbs (Gálvez-García et al., 2020). Using three different tasks, i.e., lexical decision, sound judgment, and action judgment, for concrete nouns with high and low sound or action features, a recent fMRI study provided neuroimaging evidence indicating that the retrieval of a certain feature and the engagement of modality-specific perceptual–motor areas in conceptual processing strongly depended on task demands; this was based on the result that neural activations for action and sound features of concepts were selectively recruited in motor- and auditory-related judgment, respectively (Kuhnke et al., 2020).

Along with the findings above, this study suggests that the reactivation of motor experiences in abstract verb processing is also task-dependent. Only tasks that involve the retrieval of the motor feature of abstract verbs may activate the motor system. In the test phase of Experiment 1b, we used the word font color judgment task, which might only require superficial word processing of surface characteristics, such as font color. Hence, the motor features of abstract verbs were not likely to be retrieved. However, in Experiments 1a and 2, the tasks demanded deep semantic processing, which might have included the retrieval of motor features of abstract verbs.

Our results are in line with those of a study investigating the embodied congruency effect in a font color judgment task for concrete words related to spatial position (e.g., *sky/base*; Lebois et al., 2015). In three experiments, Lebois et al. (2015) found that when participants were unaware of the spatial features of words from the initial color task instructions, a congruency effect between word verticality (high/low) and the response direction (up/down) failed to emerge, even when there were three catch trials in the task to ensure semantic processing. However, when participants were told about the verticality of the words before the color task, or an explicit semantic task (i.e., to judge the word’s meaning was spatial feature-related/concrete/abstract or not) was added prior to the color task, the congruency effect could be observed. These results proved that grounded features in a concept become active only when the task makes them salient. Even when the grounded features are central to a word’s meaning, their activation still depends on the task. However, using the same color task, our results deviated from Öttl et al.’s (2017) study. Öttl et al. (2017) asked the participants to learn novel words with novel object referents by interacting with them in the learning phase and found a congruency effect in the word font color judgment task in the test phase, suggesting that motor areas in the brain were active during the processing of words that had previously been associated with sensorimotor experiences. It is noteworthy that the novel words in this study referred to the names of the concrete entities on either the upper or lower position of the wall and were learned by participants holding a name card with one hand and pointing and touching the object entity with the other hand, while listening to the experimenter’s verbal introduction of the name (e.g., *this is NIGE*). Therefore, there might be a variety of sensorimotor experiences encoded during the learning phase, including the visual–spatial experiences, the action experiences of moving arms or moving eyes upward and downward, the tactile experiences of touching the object in the upper or lower position, and so on. Thus, the congruency effect in the color task may result from any one of them being reactivated. To date, a series of experiments by Günther et al. (2018, 2021) could not find an action congruency effect in the font color judgment task with novel words learned in a pure linguistic context independent of any sensorimotor experience. Future research should further investigate other factors that might influence the involvement of the motor system in word processing.

When interpreting the present data, several limitations should be considered. First, the ACE of Experiment 1a was obtained by the simple effect analyses without a significant interaction, which somewhat reduced the statistical power of our results. Second, the process of learning novel words in Experiment 2 might resemble the process of second language learning based on bilingual wordlists, which was, to some extent, different from the meaning learning of a real word. Using familiar abstract verbs as interpretive words did not prevent participants from learning novel words using the meaning association strategy. Third, factors, such as attentional factors (Santiago et al., 2012), which might influence the conceptual congruency effect, including the ACE, deserve more attention in future. Furthermore, the reliability of the embodied effect we used, that is, ACE, has recently been questioned by some researchers (Morey et al., 2021). fMRI research in future is needed to examine the neural changes in the motor brain areas after an increase in motor features of abstract words and to provide more convincing evidence for the causal role of motor features in the modality-specific representations of abstract concepts.

In conclusion, this study provides evidence of the causal link between the predominance of motor features of abstract verbs and their representations in the motor system. Additionally, we found that the reactivation of motor experiences in abstract verb processing was task-dependent. Our findings support embodied views that assume that representations of abstract concepts are grounded in sensorimotor experiences.

## Data availability statement

The datasets for this study can be found in the Supplementary material.

## Ethics statement

Ethical review and approval was not required for the study on human participants in accordance with the local legislation and institutional requirements. The patients/participants provided their written informed consent to participate in this study.

## Author contributions

XL and HJ contributed to the design of the study and manuscript drafting and revision. XL, DL, and CW executed the experiments. XL, HJ, and YX analyzed the data. All authors contributed to the data analysis and manuscript revision and approved the submitted version.

## Supplementary material

Supplementary Material 1 Materials of the experiments.

Supplementary Material 2 Raw data of the experiments.
